# Supplementary material for: Variability in the location of high frequency oscillations during prolonged intracranial EEG recordings
Source: Nat Commun. 2018 Jun 1;9:2155. doi: 10.1038/s41467-018-04549-2 (PMC5984620; doi:10.1038/s41467-018-04549-2)
Supplement: Supplementary file 1 — Supplementary Information [file 41467_2018_4549_MOESM1_ESM.pdf]

Variability in the location of High Frequency Oscillations during prolonged intracranial EEG recordings

Gliske et al.

Supplement

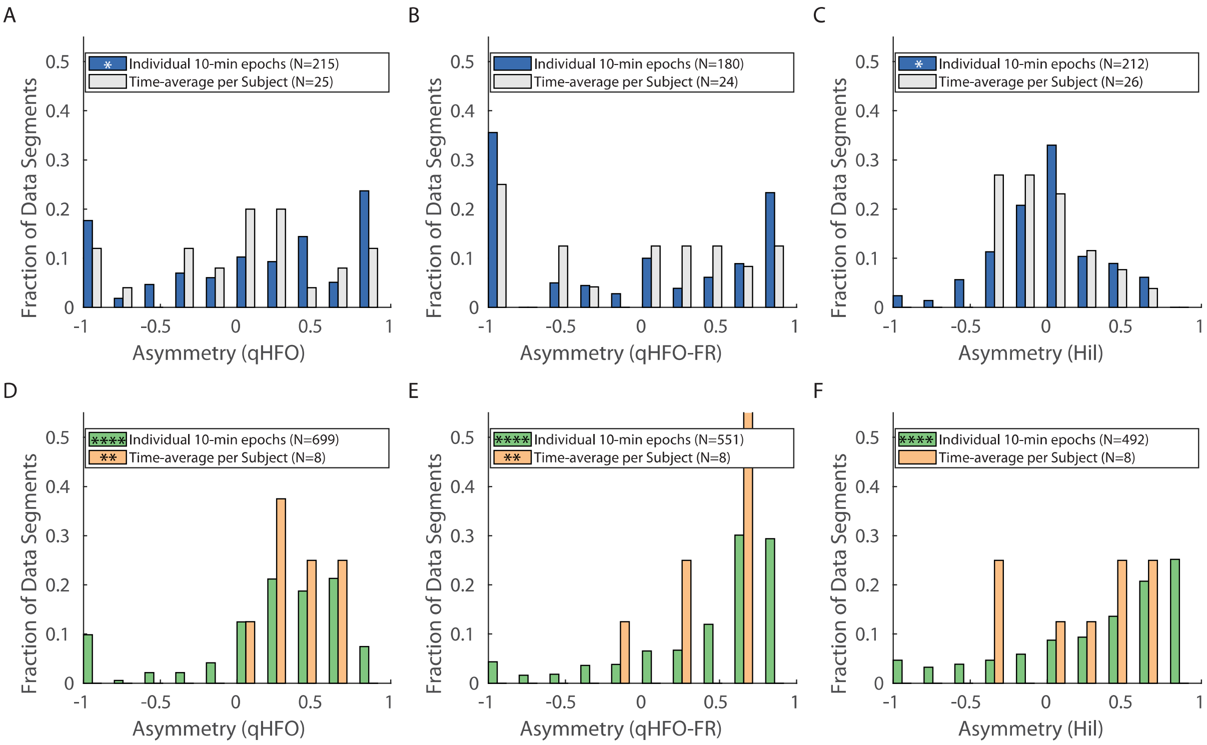

Supplementary Figure 1: Asymmetry values with respect to the clinically determined seizure onset zone (SOZ) for good surgery outcome subjects. The plot is organized as in Fig. 1 and represents a subset of that data corresponding to subjects with good surgery outcomes (ILAE Class I). (A-C) Mayo Cohort; (D-F) UM Cohort, NREM data; (A,D) qHFO detector; (B,E) fast ripple enriched qHFO detector; (C,F) Hilbert-transform based HFO detector. Statistical significance of the median being positive (one-sided Wilcoxon Sign Rank test) of each individual histogram is shown with asterisks on the colored box in the legend: * $p < 0.05$, ** $p < 0.01$, *** $p < 0.001$, **** $p < 0.0001$. The time average per subject asymmetries tends to reach statistical significance much less often than the individual 10-minute asymmetries due to the lower number of data points per histogram

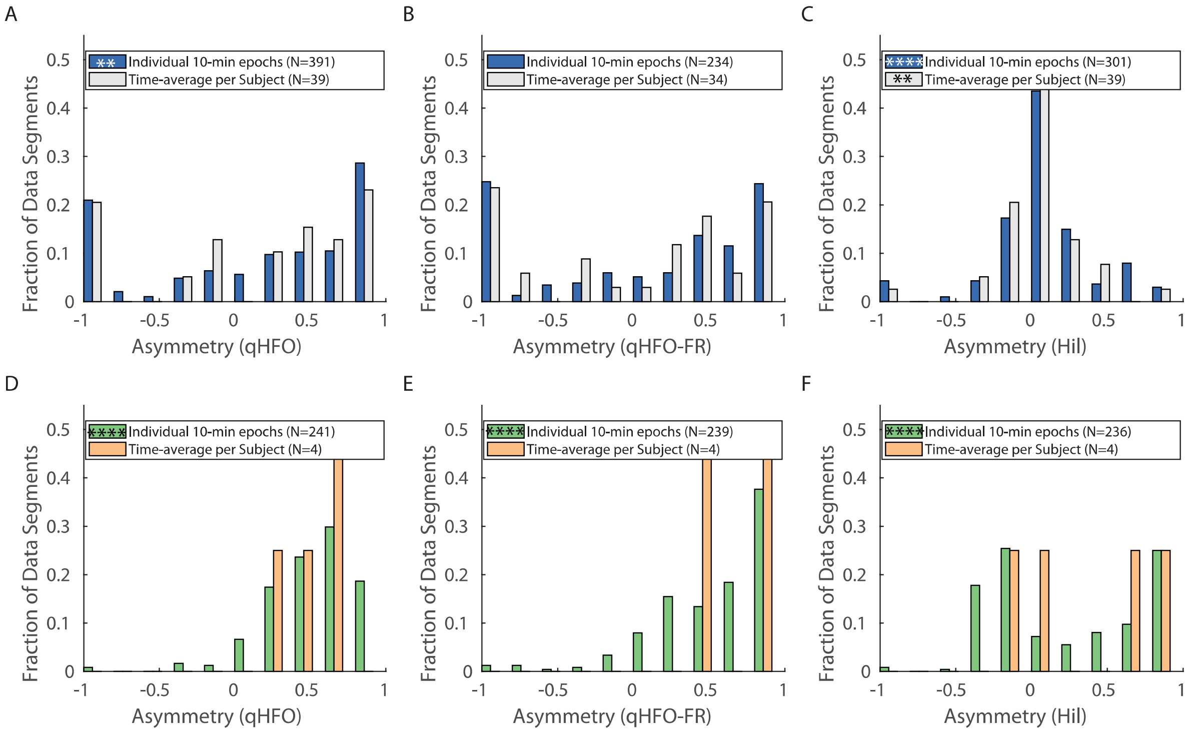

Supplementary Figure 2: Asymmetry values with respect to the clinically determined seizure onset zone (SOZ) for poor surgery outcome patients. The plot is organized as in Fig. 1 and represents a subset of that data corresponding to subjects with poor surgery outcomes (ILAE Class II-IV). (A-C) Mayo Cohort; (D-F) UM Cohort, NREM data; (A,D) qHFO detector; (B,E) fast ripple enriched qHFO detector; (C,F) Hilbert-transform based HFO detector. Statistical significance of the median being positive (one-sided Wilcoxon Sign Rank test) of each individual histogram is shown with asterisks on the colored box in the legend: * $p < 0.05$, ** $p < 0.01$, *** $p < 0.001$, **** $p < 0.0001$.

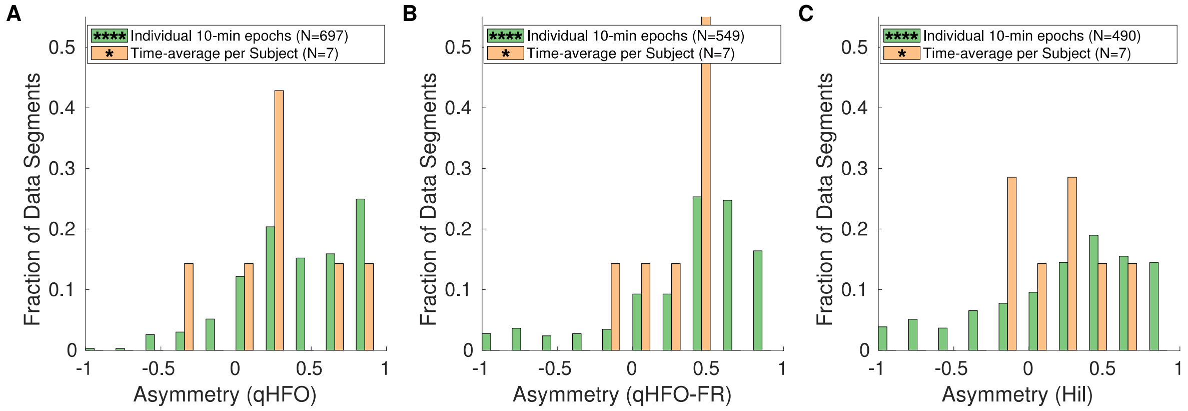

Supplementary Figure 3: Asymmetry values with respect to the resected volume (RV) for good surgery outcome subjects. (A-C) UM Cohort, NREM data. Similar to Supplementary Fig. 1 (D-F), but the HFO asymmetries are computed with respect to the resected volume, as determined by discussion with the attending neurosurgeons, for patients with good outcome. Only the UM Cohort had RV information available. (A) qHFO detector; (B) fast ripple enriched qHFO detector; (C) Hilbert-transform based HFO detector. Statistical significance of the median being positive (one-sided Wilcoxon Sign Rank test) of each individual histogram is shown with asterisks on the colored box in the legend: * $p < 0.05$, ** $p < 0.01$, *** $p < 0.001$, **** $p < 0.0001$.

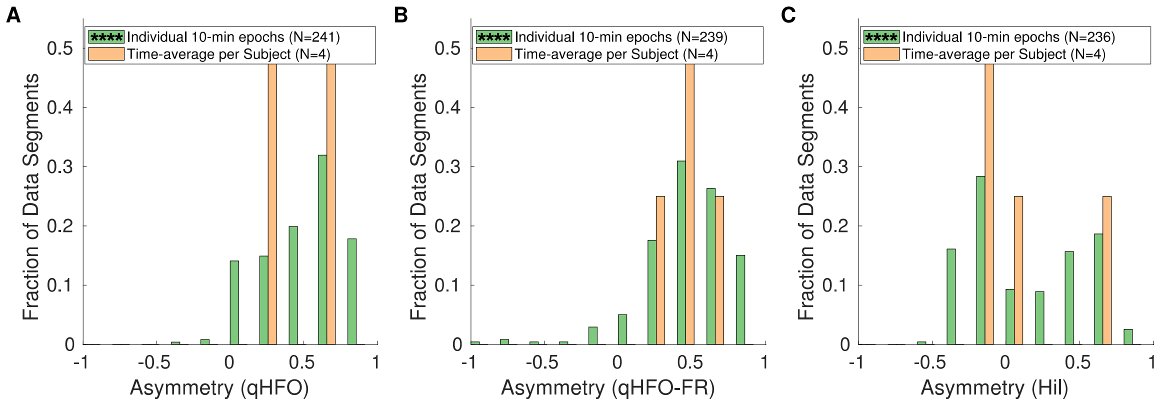

Supplementary Figure 4: Asymmetry values with respect to the resected volume (RV) for poor surgery outcome patients. (A-C) UM Cohort, NREM data. Similar to Supplementary Fig. 1 (D-F), but the HFO asymmetries are computed with respect to resected volume for ILAE Class II-V patients. Statistical significance of the median being positive (one-sided Wilcoxon Sign Rank test) of each individual histogram is shown with asterisks on the colored box in the legend: * $p < 0.05$, ** $p < 0.01$, *** $p < 0.001$, **** $p < 0.0001$.

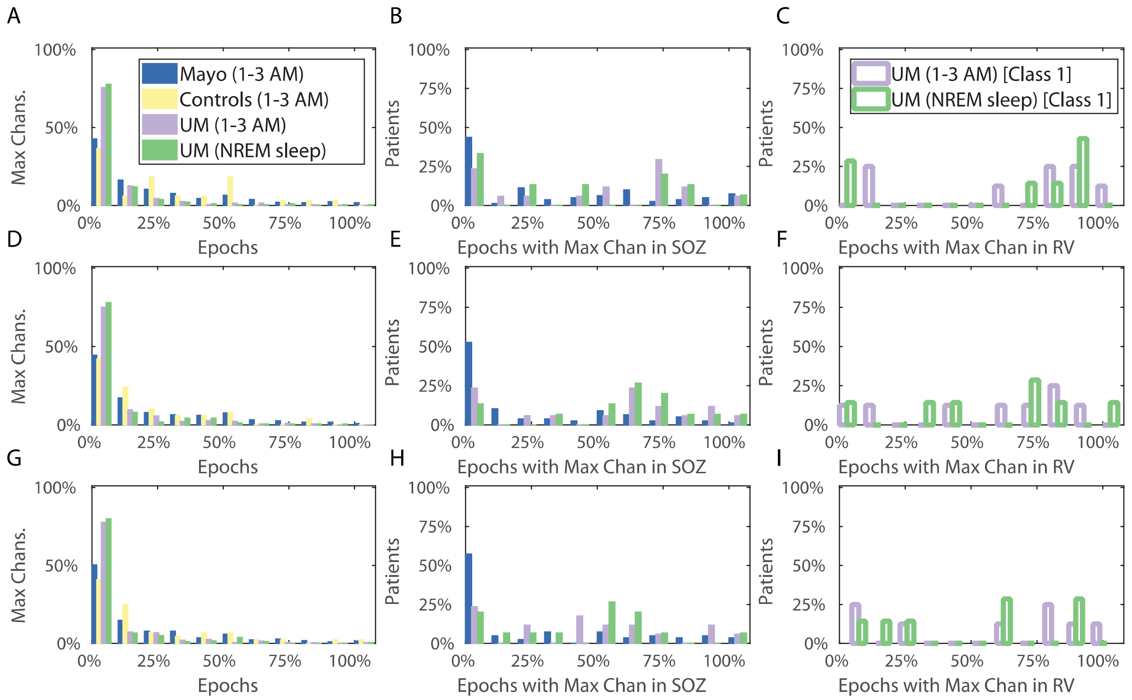

Supplementary Figure 5: Variation in the channel with maximum HFO rate per epoch. Results are shown for the qHFO detector (A,B,C), the fast ripple enriched qHFO detector (D,E,F) and a Hilbert-transform based HFO detector (G,H,I). The first column (A,D,G) shows the distribution of how often each channel has the maximum rate. Values are normalized to the number of channels per cohort, and channels which never have the maximum HFO rate in any epoch are ignored. Second column (B,E,H) contains the distribution of what fraction of time (epochs) the channel with the maximal rate is within the seizure onset zone (SOZ), normalized to the number of patients per cohort. The third column (C,F,I) is similar to the second column (B,E,H) but compares with respect to the resected volume (RV) instead of SOZ, and restricts to patients with ILAE Class I outcomes. Overall, many channels have the highest HFO rate in only a few epochs. Also, a large fraction of patients typically have the channel with maximum HFO rate in a given epoch outside the SOZ or RV. Both observations suggest high temporal variability in the HFO rates.
